# Measurements of the Depth of Sleep

**Published:** 1885-05

**Authors:** 


### Measurements of the Depth of Sleep.

Two of Vierordt's pupils, Moninghoff and Piesbergen, have made the depth of sleep the subject of an investigation. (*Science.*) They worked upon the principle that the depth of sleep is proportional to the strength of the sensory stimulus necessary to awaken the sleeper, that is, to call forth some decisive sign of awakened consciousness. As a sensory stimulous they made use of the auditory sensation produced by dropping a lead ball from a given height. The strength of the stimulous was reckoned, in accordance with some recent investigations of Vierordts, as increasing, not directly as the height, but as the 0.50 power of the height. For a perfectly healthy man, the curve which they give shows that for the first hour the slumber is very light; after one hour and fifteen minutes the depth of sleep increases rapidly, and reaches its maximum point at one hour and forty-five minutes: the curve then falls quickly to about two hours and fifteen minutes, and afterwards more gradually. At about four hours and thirty minutes, there is a second small rise which reaches its maximum at five hours and thirty minutes, after which the curve again gradually approaches the base line until the time of awakening. Experiments made upon persons not perfectly healthy, or after having made some exertion, give curves of a different form.—*Medical Advocate.*

THE conveniences of our modern inventions are to some extent offset by the draw-backs. For instance, the telephone with the mischievous girl at the central office. A secular contemporary reports the following case: A husband calls upon a Dr. and tells him that his wife has "a severe pain in the back of the neck, and complains of a sort of goneness in her stomach." "She has malarial colic," returns the man of medicine. "What shall I do for her?" asks a now anxious husband. The girl at the central now switched on a machinist who was talking with a saw-mill man about his boiler, and this is the advice which falls on the husband's tympanum: "I think she's covered with scales inside about an inch thick. Let her cool down during the night, and before she fires up in the morning take a hammer and pound her thoroughly all over, and then take a hose and hitch it to a fire-plug and wash her out." The doctor and the husband do not now speak as they pass by, and the doctor has found the telephone too expensive an instrument for further continuance.—*Medical Age.*

